# Measures of Clonal Hematopoiesis: Are We Missing Something?

**DOI:** 10.3389/fmed.2022.836141

**Published:** 2022-03-31

**Authors:** Leonid V. Bystrykh, Mirjam E. Belderbos

**Affiliations:** ^1^Department for Stem Cell Biology and Ageing, European Research Institute for the Biology of Ageing, University Medical Center Groningen, Groningen, Netherlands; ^2^Princess Máxima Center for Pediatric Oncology, Utrecht, Netherlands

**Keywords:** hematopoiesis, clone, stem cell, clonal diversity, ageing, lineage tracing

## Abstract

Clonal Hematopoiesis (CH) is a common, age-related phenomenon of growing scientific interest, due to its association with hematologic malignancy, cardiovascular disease and decreased overall survival. CH is commonly attributed to the preferential outgrowth of a mutant hematopoietic stem cell (HSC) with enhanced fitness, resulting in clonal imbalance. In-depth understanding of the relation between HSC clonal dynamics, CH and hematologic malignancy requires integration of fundamental lineage tracing studies with clinical data. However, this is hampered by lack of a uniform definition of CH and by inconsistency in the analytical methods used for its quantification. Here, we propose a conceptual and analytical framework for the definition and measurement of CH. First, we transformed the conceptual definition of CH into the CH index, which provides a quantitative measure of clone numbers and sizes. Next, we generated a set of synthetic data, based on the beta-distribution, to simulate clonal populations with different degrees of imbalance. Using these clonal distributions and the CH index as a reference, we tested several established indices of clonal diversity and (in-)equality for their ability to detect and quantify CH. We found that the CH index was distinct from any of the other tested indices. Nonetheless, the diversity indices (Shannon, Simpson) more closely resembled the CH index than the inequality indices (Gini, Pielou). Notably, whereas the inequality indices mainly responded to changes in clone sizes, the CH index and the tested diversity indices also responded to changes in the number of clones in a sample. Accordingly, these simulations indicate that CH can result not only by skewing clonal abundancies, but also by variation in their overall numbers. Altogether, our model-based approach illustrates how a formalized definition and quantification of CH can provide insights into its pathogenesis. In the future, use of the CH index or Shannon index to quantify clonal diversity in fundamental as well as clinical clone-tracing studies will promote cross-disciplinary discussion and progress in the field.

## Introduction

The dual ability of adult stem cells to self-renew and to produce all mature cell types is unique and underlies tissue homeostasis, growth and (at least to some extent) tissue regeneration. Dysfunction of tissue stem cells is thought to underlie several diseases, including hematopoietic failure and cancer (1–4). Accordingly, assessing the number of stem cells and their clonal contribution to tissue generation has been subject of scientific interest and controversy for decades (5–8).

Clone tracking has been used most extensively in the hematopoietic system, in part due to the relative ease of sampling and potential for longitudinal assessment. Pioneering studies have used various clonal markers, such as viral integration sites, DNA barcodes, transposons, mitochondrial DNA, X-chromosome silencing, fluorescent tags or naturally occurring somatic mutations, to trace hematopoietic stem cells (HSCs) in murine xenografts, monkeys and humans (9–17). These studies estimate that, at steady-state, human hematopoiesis is supported by hundreds of thousands of HSCs (15, 16). In a healthy individual, at any moment in time, the majority HSCs are assumed to contribute more or less equally to hematopoiesis, albeit their exact numbers and lineage choices remain controversial (11, 12, 16, 17). Deviations from this “clonal equilibrium” are considered to result from heterogeneity in the competitive fitness of the parental HSCs. Such heterogeneity may result from the age-related accumulation of damage in HSCs and may predispose to several hematologic diseases (15, 16).

The interest in HSC clone tracing has risen dramatically by the recent discovery of clonal hematopoiesis (CH). CH is defined as the detectable (above some arbitrary threshold) presence of cancer-associated somatic mutations in an apparently healthy blood system (18, 19). CH is present in >10% of individuals older than 70 years and is associated with increased risk of hematologic malignancy, cardiovascular disease and all-cause mortality (18–20). Nonetheless, the absolute risk of hematologic cancer in individuals with CH is low, and there is a need to identify features of CH that predict leukemic progression versus those that do not.

To better understand the relation between HSC clonal dynamics, CH and its progression toward hematologic malignancy, it will be useful to integrate knowledge from fundamental lineage tracing studies with clinical epidemiological data on CH. However, to allow for such integration, several issues need to be addressed. While the definition of the term “clone” is intuitively well understood, the term “clonal hematopoiesis” is less clear. In cell biology, the term “clone” refers to a population of cells that are derived from the same ancestor, which can be identified by the presence of certain unique, heritable markers (6, 9). In clinical studies, the term “CH” refers to the presence of one or a few driver mutations with a variant allele frequency of at least ∼2%, while the overall number of HSC clones and their relative sizes remain unreported (18, 19). As a result, the processes and dynamics that underlie CH in mice as well as in humans are difficult to compare and reconcile.

Here, we make an attempt to further formalize the definition of CH and determine the direct consequences of such a definition. This attempt employs markers of population diversity used to quantify the abundance of CH clones within a heterogeneous HSC population. We propose that this approach will allow for a more reliable, quantitative measurement of CH, which can be applied to different markers or platforms, in model organisms as well as in humans.

## Materials and Methods

### *In silico* Simulation of Clonal Skewing and Richness

**FIGURE 1:**
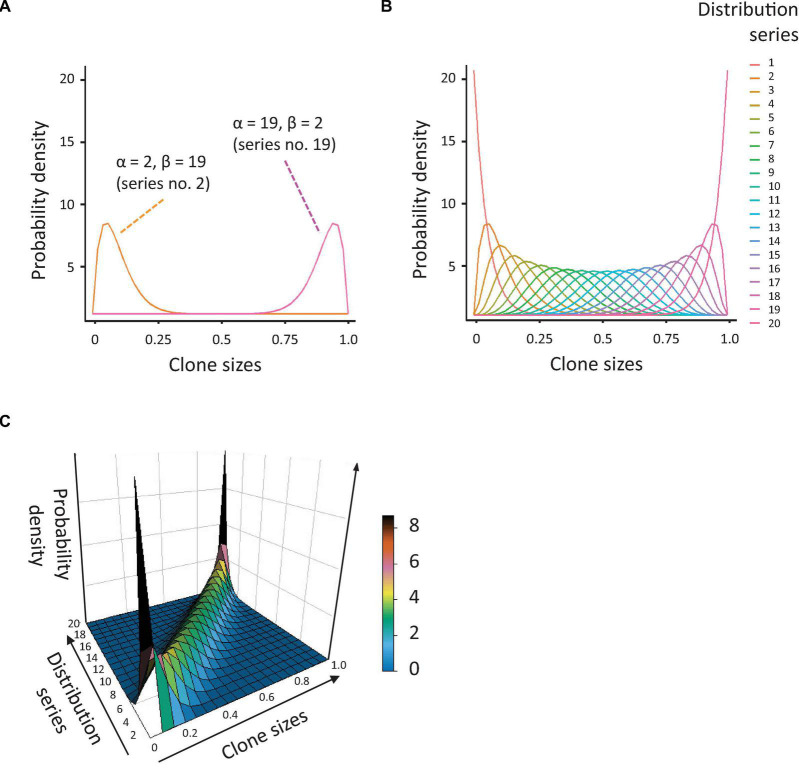
Simulation of population diversity based on the beta distribution. **(A)** Probability density distributions, based on the beta distribution, generated by reciprocal variation of the alpha and beta coefficients. The orange curve represents the distribution for alpha = 2 and beta = 19 (many small clones and a few big), the pink curve is the distribution for alpha = 19 and beta = 2 (many large clones and a few small). **(B)** Overview of all probability distributions tested. Note that these vary from highly skewed to near-normal. **(C)** 3-dimensional depiction of the probability distributions shown in Figure 1B.

To allow *in silico* quantification of CH, we first generated a set of synthetic data with enough diversity in clone sizes and distributions. Out of the known data distributions, we decided to use a beta distribution, with which it is relatively easy to generate datasets of variable skewing. We generated a series of 20 beta distribution profiles with alpha and beta coefficients changing reciprocally, from 1 to 20 and 20 to 1, respectively. In this way, we obtained datasets of variable skewing, ranging from an extremely left-shifted mean with right-skewed tail of the distribution (many very small values and a few big, which is the case in a highly (mono-)clonal distribution), to an extremely right-shifted mean with left-skewed tail (many big values and only a few small, Figures 1A,B). We combined this series of beta distributions with a dataset of populations with diverse clonal richness, ranging from 50 to 500 data values (clones) with steps of 25 (19 populations in total). The resulting array of data (with diverse skewing and richness, Figure 1C) was used to calculate several indices of population diversity and equality: Shannon, Simpson, Gini, and Pielou, as described below (and obviously the CH index or *I_*CH*_*, after we defined it).

### Population Richness

Population richness is defined as the simple number of clones in a given sample. This is also known as the nominal count. The more clones, the ‘richer’ the sample. Population richness does not take into account the relative size of each clone. Thus, the richness of a sample is as much affected by a small clone as by a very large clone.

### Shannon Diversity Index

Diversity of any population is not only determined by the number of clones, but also by their sizes. An elegant solution to quantify the diversity of such a population is the *Shannon diversity index*, which is also known as the Shannon-Wiener index of simply the Shannon index *H*_*Sh*_:


$$H_{S\,h}=\sum_{i=1}^{N}p_{i}\,l\,n\,\left(p_{i}\right)$$

where, *p*_*i*_ is the relative abundance of each *i*th clone and *N* is the maximum number of clones in the sample. Notably, although most studies use the natural logarithm, the base of the logarithm used to calculate the Shannon index can be chosen freely, and commonly used bases include logarithm base 2 and 10 (scikit-bio uses variable base, default base in vegan (R) is exponent). If all clones are of equal size, then *p_*i*_ = 1/N* and *H*_*Sh*_ is maximal. If clone sizes are not equal, then for each clone, *p*_*i*_ reflects its abundance divided by the total abundance of all other clones.

A remarkable feature of the Shannon index is that it can be used to predict the number of clones (a.k.a. richness) *C*, while taking into account their relative sizes:


$$C_{S\,h}=\text{exp}\,\left(H_{S\,h}\right)$$

The Shannon index is particularly useful in datasets with large numbers of small clones/sequencing noise, as the contribution of these clones to the overall diversity will be far lower than the contribution of the larger clones. Therefore, the Shannon index is relatively unaffected by (arbitrary) thresholds for clonal detection and by low-frequency noise.

### Simpson Index

The Simpson index (λ) is usually seen as alternative to the Shannon index, and is defined as follows:


$$\lambda =1-\sum_{i=1}^{N}p_{i}^{2}$$

Similar to the Shannon index, the Simpson index is maximal when all clones are of the same size, and equal to zero when only one clone is present.

### Pielou Index

To quantify evenness in clonal sizes, we can use the Pielou index, also known as the Pielou’s evenness index (21). The Pielou index *J* can be calculated from the Shannon index and is defined as follows:


$$J=\frac{H_{S\,h}}{H_{m\,a\,x}}$$

where, *H*_*Sh*_ is the Shannon diversity index and *H*_*max*_ is the maximum possible value of *H* (i.e., if every clone is of equal size). If all clones in a sample are of equal size, then *J* = 1. If there is a very strong bias (i.e., one very big clone), *J* is close to zero.

### Gini Index

Finally, the *Gini index* (also known as Gini coefficient) provides an alternative method to quantify the clonal inequality of a sample. The Gini index is commonly used in economics, as a measure of inequality of income or wealth. It is defined mathematically *via* the Lorenz curve, which plots the cumulative frequency of a ranked population. The line at 45 degrees represents a perfectly equal distribution in frequencies. In terms of clones, all clones are of equal size. The less equal the data (the more difference in clone sizes), the more the Lorenz curve deviates from the perfect diagonal. The Gini index *G* is derived by comparing these curves, and is defined as:


$$G=\frac{A}{A+B}$$

where, A is the area between the perfect diagonal and the Lorenz curve, and B is the area below the Lorenz curve. The Gini index can range from 0 (perfect equality, all clones are of equal size) to 1 (perfect inequality, one very large clone).

### Programming Tools

All simulations were made in Python 3 on a regular laptop, using standard python libraries: *SciPy*, *Numpy*, and the *Scikit-bio* package (22–24). The beta distribution was generated using *Scipy.stats* (*scipy.stats.beta())* and beta random sampling with *numpy.random.beta()* from the *numpy* package. All diversity and (in)equality indexes are from *skbio.diversity.alpha*. In parallel, the same simulations were done in R, using core functions (beta distribution, *dbeta()*, *rbeta()*) and the following packages for population indexes: *vegan* (25), *reldist* (26) and *OTUtable* (27). For linear models, the python Statsmodels package was used in python (*olm()*). In R the lm() core function was employed. All scripts can be found on GitHub: https://github.com/LeonidBystrykh/Measures_of_CH.

## Results

### Formalizing the Conceptual Definition of Clonal Hematopoiesis Into the CH-Index

A screen through the literature dedicated to CH in humans provides lots of details regarding gene mutations, possible biological effects etc. Surprisingly, it is difficult to trace a quantitative and well-defined measure of the CH. Therefore, we decided to (re)define it here, namely we first formalized the conceptual definition of CH into a quantitative measure of clone numbers and sizes.

Assuming a unimodal distribution of clone sizes, the clonal composition of any sample can be seen as the sum of the number of clones and their relative sizes. In other words, for each clone I with relative size *x_i_* in the bone marrow (or any other stem cell compartment) and amplification factor (relative contribution to hematopoiesis)*a_i_*, the resulting clone size in blood will be *a_i_x_i_*. For the entire population of clones, their contribution, as measured in blood can be quantified as:


$$C_{C\,H}=\sum_{i=1}^{n}a_{i}\,x_{i}$$

In reality, during normal hematopoiesis, if we measure clones and their sizes in blood, most of the time we do not know the values of HSC sizes, *x*, nor their relative contribution coefficients *a* in separate, rather we measure the resulting value *a_i_x_i_* for any clone *i*. If we deal with retro- or lentivirally transduced stem cells (like in mouse models or gene therapy trials) (9, 10, 13, 28, 29) then estimation of these parameters becomes theoretically possible.

In clinical studies on CH, not all clones in the blood, nor in the bone marrow are analyzed (18–20, 30). Rather, these studies rely on the detection of one or a few clones with somatic mutations above a critical threshold for detection. Depending on the sensitivity of the applied sequencing technique, this threshold can vary from 2%, or 0.5%, or anything in between (19, 20, 31). Consequently, we also introduced a threshold for detection into the model, and defined the CH index (*I*_*CH*_) as the fraction of all blood cells, which carry a genetic mark with a frequency above this minimal threshold. Therefore, clonal index will be a sum of all clonal contributions for clones:


$$I_{C\,H}=\sum_{i=1}^{n}a_{i}\,x_{i}$$

$$\text{for}\,a_{i}\,x_{i}>t\,h\,r\,e\,s\,h\,o\,l\,d$$

where, *x_i_* and *a_i_* have the same meaning as above, but the index *I*_*ch*_ counts only clonal contributions above the relevant threshold. In most studies, clone size is expressed as a fraction of the total count of all alleles. Therefore *I*_*CH*_ is expressed as a fraction of the total read counts for each particular mutation detected. As an example, for a sample in which a single clone passes the threshold for detection with a contribution coefficient *a* of 0.2, the clonal index will be 0.2. If a sample contains two detectable clones, of which one with *a* = 0.15, and another with *a* = 0.05 (both above arbitrary threshold), the clonal index will be 0.15+0.05 = 0.2. Note that *I*_*CH*_ varies between 0 and 1, where 0 represents case of a “normal” hematopoiesis (no big clones detected), and 1 represents an extreme case of a clonal hematopoiesis with profound dominance of a few clones. To generate sufficient variation, we selected a threshold of I_*CH*_ of 0.005 for the simulations described in this manuscript. Repeated analyses, using alternative thresholds of 0.002 or 0.02, did not affect our conclusions.

### Simulation of Clonal Distributions

Next, we aimed to assess the capacity of the *I*_*CH*_, as well as several existing indices of clonal diversity, to detect and quantify CH. To this aim, we generated an array of data, based on the beta distribution, representing populations with different degrees of skewing and richness (Figures 1A–C, details in Methods). As visualized in Figure 1, the resulting distributions demonstrated a large variability in the degree of skewing. When alpha <beta, the means of the distributions were shifted to the left (many small values and a few big). When alpha and beta are equal, the resulting distributions were close to normal. When alpha >beta, the means of the distributions were shifted to the right (many big values and a few small). The former dataset reflects a strong clonal bias, whereas the latter has the lowest bias.

### Quantifying Clonality by the CH Index

**FIGURE 2:**
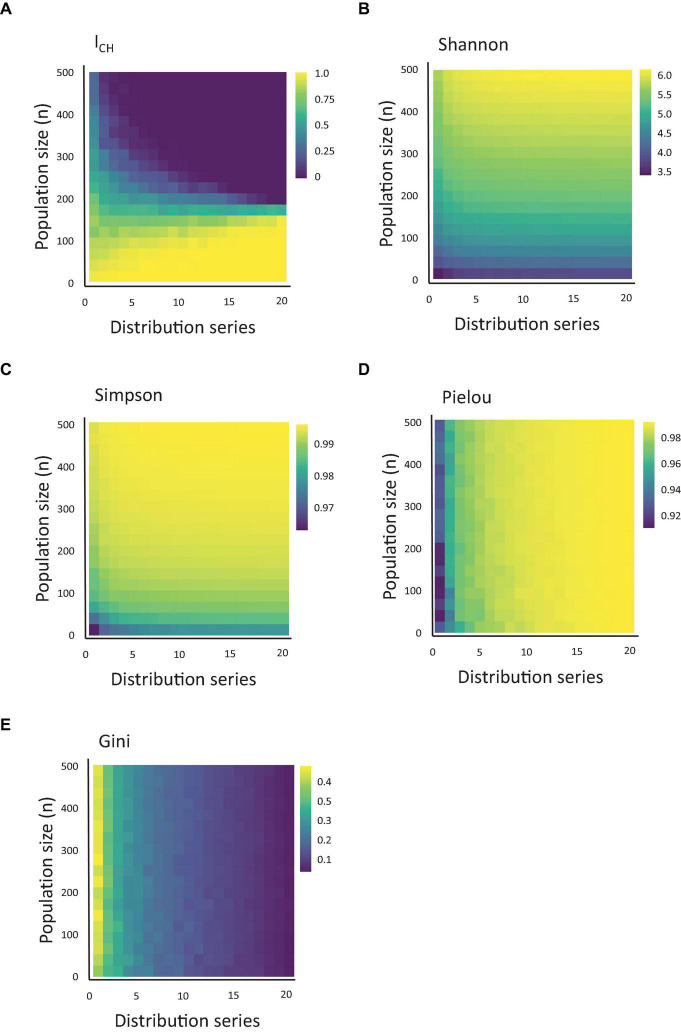
Quantification of CH using measures of population diversity. **(A)** Heatmap of the value of *I*_*CH*_, depending on the clonal population size and population skewing, simulated using the series of 20 distributions shown in Figure 1 (starting from the lowest alpha and highest beta) and using a threshold of 0.005. **(B–E)** The same as Figure 1A, measured for Shannon, Simpson, Pielou and Gini indexes.

For each combination of beta distribution and population richness (), we calculated the *I*_*CH*_ index (Figure 2A). As described in the Methods section, this index represents the sum of all observable clones and their relative sizes above the arbitrary threshold. We noted that the *I*_*CH*_ index responded both to variations in population skewing and richness, and that the impact of each parameter depended on the value of the other. At very low levels of population richness, the *I*_*CH*_ remained non-zero at any degree of population skewing. To the contrary, at high levels of population richness, *I*_*CH*_ was relatively stable and close to 0.

### Other Measures of Diversity

Next, we asked how the *I*_*CH*_ index compares to other population indexes used in the biological literature. Therefore, for the same datasets used to calculate *I_*CH*_*, we calculated the values of the Shannon, Simpson, Gini and Pielou index (Figures 2B–E). Interestingly, similar to *I_*CH*_*, both the Shannon and Simpson indexes also responded to variation in clonal sizes (a.k.a. richness) and skewing, but each in its own way (and differently from *I*_*CH*_). While *I*_*CH*_ decreased upon increased population skewing or richness, the Simpson and Shannon indexes increased at similar conditions. Remarkably, the Gini and Pielou indexes showed a stark difference in their responses. These indexes were practically insensitive to variations in population richness and responded almost solely to variations in population skewing.

### Can One Index Predict the Value of Another?

**FIGURE 3:**
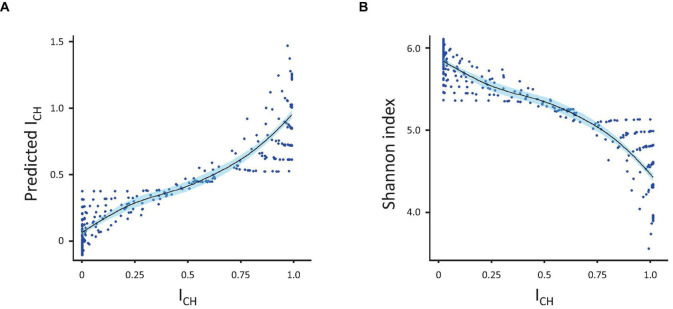
Prediction of *I*_*CH*_. **(A)** Correlation between the measured and predicted (by linear regression) values of *I*_*CH*_. **(B)** Inverse correlation between *I*_*CH*_ and the Shannon index. Correlations were calculated using the population diversity datasets presented in Figure 1.

As each index responded to variations in skewing and richness differently, we subsequently tested whether the CH-index could be explained (predicted) by any combination of the other four indexes. For this, we used an ordinary linear model, which allowed us to see the fraction of the variation explained as well as to assess which individual index contributed the most to the model (Figure 3). Analysis showed that Shannon and Simpson indexes, alone or in combination, explained the greater part of the *I*_*CH*_ (R-squared 0.83 for the Shannon index only, 0.594 for the Simpson index only and 0.882 for both indexes together). In contrast, the Gini and Pielou indexes together explained only 0.092 (R-squared) of the variance. When added to the Shannon and Simpson indexes, the Gini index only added 0.05 to the explained variance (R-squared improved from 0.883 to 0.888). The same happened if Pielou index was added to the equation, instead of the Gini index. Adding a fourth index (either Gini or Pielou) did not improve the model further.

To conclude, this study indicates that the *I*_*CH*_ index, as it is formalized above, can be best quantified using indexes of population diversity, in particular the Shannon diversity index. In the future, more indexes can be tested and more analysis will be needed to better understand what *I*_*CH*_ actually is in terms of population theory, which currently does not include the *I*_*CH*_.

## Discussion

While our understanding of the pathogenesis and clinical consequences of CH is gradually increasing, a few key elements are still missing. In particular, uniform quantification of CH is needed to avoid misunderstandings in the field. However, currently, a commonly accepted quantitative measure of CH is missing, and the characteristics of such a measure remain unclear. In this manuscript, we translated the conceptual definition of CH into a mathematical measure (*I*_*CH*_) of clone numbers and sizes. Subsequently, we used this measure to test the behavior of several existing indices of population diversity and (in)equality. Our results imply that, while talking about CH, we refer to a loss of population diversity. In the current simulations, we demonstrate that CH can be achieved by an increased skewing in clone sizes as well as by a decreased number of clones. Therefore, in real life, CH may not always reflect dominance of a clone with a higher and biased contribution; it might also be a matter of a reduced number of stem cells.

The dual nature of the *I*_*CH*_ presented in this paper implies that the etiology of CH may be of a dual kind as well. On the one hand, the most commonly scenario describes CH as the result of a gradual, age-dependent acquisition of somatic mutations in hematopoietic progenitor cells (19, 32–35). Once one or a few of these mutations confer a competitive advantage, the clonal mass of the cells that carry these mutations increases. As the number of mutations in stem cells increases gradually with age, so does the risk of CH (15, 16). On the other hand, a reduction in the overall number of stem cells may cause a similar effect, resulting in an apparent increase in the relative size of the remaining clones. Notably, these two scenarios are not mutually exclusive and likely coexist, even within the same patient.

These dual scenarios have several consequences. First, they provide an additional explanation for the increased prevalence of CH in stem cell transplantation recipients (13, 36–39). As these patients, as well as patients undergoing gene therapy, are transplanted with only a fraction of the donor stem cell pool, the clonal mass of each stem cell is likely higher compared to healthy age-matched controls. Notably, we recently demonstrated that the mutational consequences of transplantation on the donor stem cells are generally negligible, suggesting that clonal stem cell reduction may be particularly important in this setting (40). Second, a similar phenomenon may apply to CH in patients undergoing stem cell cytotoxic therapies (41, 42). In addition to the DNA damage inflicted by these therapies, they may also induce stem cell loss, thereby increasing the risk of CH. To distinguish between these options, in future, it will be of interest to relate the mutational signatures induced by these agents to the signatures observed in post-therapy CH (43). Third, the risk of hematologic malignancy may differ between individuals with CH due to clonal dominance versus those with CH due to clonal stem cell reduction. Discriminating between these two scenarios, for instance by the type of mutation or by the distribution of clone sizes (using the *I*_*CH*_ or Shannon index), might allow for a better prediction and risk-adjusted screening of hematologic malignancies.

Application of the *I*_*CH*_ in real-life requires knowledge of both of its parameters, i.e., the contribution coefficient *a_n_* and the number of clonogenic cells, *x_n_*. Currently, these values are not exactly known and difficult to estimate experimentally, although not impossible and highly necessary. For instance, using DNA barcoding, we were able to trace major clones in blood back to HSC populations in the bone marrow (10, 44). In theory, the same possibility exists for gene therapy trials. These trials provide a unique opportunity to integrate lineage tracing of single HSCs (e.g., using integration sites, barcoded vectors, etc.) with regular analysis of clonal developments based on somatic mutations in blood. Parallel assessment of single mutation VAFs as well as overall clonality may provide insight into the origin and clinical consequences of aberrant clones, after gene therapy and during normal ageing. Furthermore, longitudinal assessment of *I*_*CH*_ (or of the Shannon index) in these individuals may allow early detection of (vector-related) malignancies.

The feasibility of integrating knowledge from HSC lineage tracing studies with somatic mutation profiles relies critically on a uniform, quantitative definition of CH. Such a definition will not only prevent miscommunication, but will also inform us what kind of experimental data are required. Although data from current publications allow for some quantification, the diversity of sequencing strategies, detection methods and thresholds for detection makes it difficult to compare and reconcile their results. Here, we propose that the Shannon (or Simpson) diversity indexes, which are well characterized and relatively threshold-free, can be used to reliably detect clonal aberrations in a population. These indexes are well-established in stem cell lineage-tracing studies in mice (10, 45–47), as well in population studies of all kinds. In theory, these indices can be applied to unfiltered (whole genome or whole exome) sequencing data. During polyclonal hematopoiesis with many small and constantly changing clones (and which are difficult to discriminate from sequencing noise), diversity will be maximal. Once one or multiple clones in the population grow systematically, this may be detected by a reduction in the Shannon or Simpson index.

Further development of the theoretical background and technical measurement of CH is needed, to stimulate experimental research and to improve our understanding of clonal developments during normal aging, gene therapy and leukemia.

## Data Availability Statement

The scripts and datasets for this study can be found on GitHub: https://github.com/LeonidBystrykh/Measures_of_CH.

## Author Contributions

LB developed the theory and performed the computations. MB provided the feedback, checked the analyses, and helped refine the theory. Both authors wrote the manuscript and made the figures.

## Conflict of Interest

The authors declare that the research was conducted in the absence of any commercial or financial relationships that could be construed as a potential conflict of interest.

## Publisher’s Note

All claims expressed in this article are solely those of the authors and do not necessarily represent those of their affiliated organizations, or those of the publisher, the editors and the reviewers. Any product that may be evaluated in this article, or claim that may be made by its manufacturer, is not guaranteed or endorsed by the publisher.
